# Conservative Treatment of a Floating Mural Thrombus in the Descending Aorta

**DOI:** 10.1055/s-0041-1736370

**Published:** 2021-10-04

**Authors:** Johannes Goedemé, Lawek Berzenji, Simon Nicolay, Jeroen M.H. Hendriks

**Affiliations:** 1 Department of Thoracic and Vascular Surgery, Antwerp University Hospital, Edegem, Belgium; 2 Department of Radiology, Antwerp University Hospital, Edegem, Belgium

**Keywords:** floating thrombus, vascular surgery, aortic arch

## Abstract

A 70-year-old woman was referred to the department of thoracic and vascular surgery after a floating mural thrombus was found in the distal aortic arch with extension into the proximal descending aorta. Imaging and clinical examination did not show signs of embolization and treatment with aspirin and low–molecular weight heparin was started. Follow-up after 2 weeks and 6 months showed a complete resolution of the aortic mural thrombus.

## Introduction

The majority of peripheral and visceral emboli are due to cardiac diseases such as myocardial infarction, atrial fibrillation, endocarditis, and heart valve prosthetics. Noncardiac causes of aortic emboli are mainly related to aneurysmal disease, atherosclerosis of the aortic wall, or paradoxical emboli of the venous circulation. 1 Furthermore, several coagulation disorders such as protein C and protein S deficiency, hyperhomocysteinemia, antithrombin-III deficiency, and elevated factor VIII levels have also been linked to mural thrombus formation in the aorta. 2 The majority of these cases present with symptoms of peripheral embolization. However, in some cases, patients can be asymptomatic with the thrombus only being detected as an incidental finding. 3 A floating thrombus in the aorta with no signs of peripheral embolization is a rare clinical finding.

## Case Presentation

**Fig. 1:**
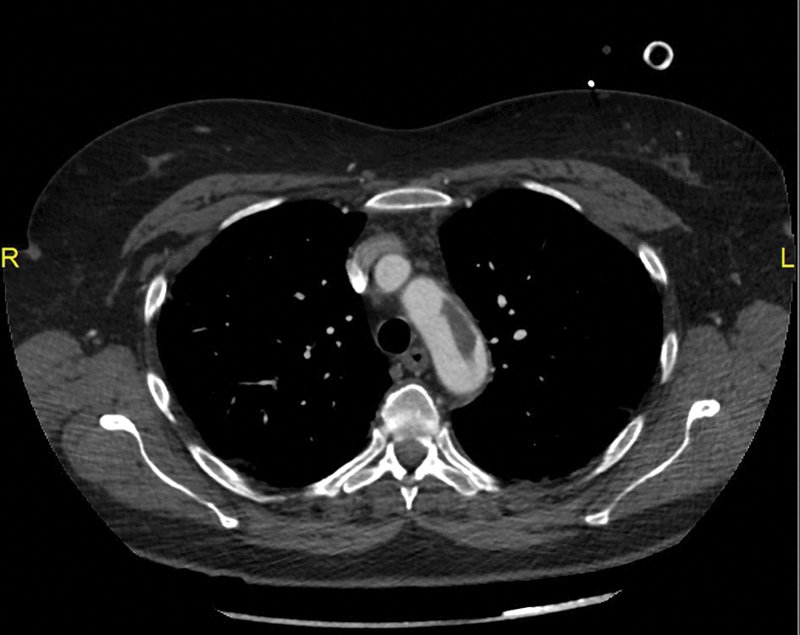
Axial arterial-phase chest computed tomography scan at the level of the aortic arch. A hypodense filling defect, consistent with a floating thrombus, can be seen in the opacified aorta.

**Fig. 2:**
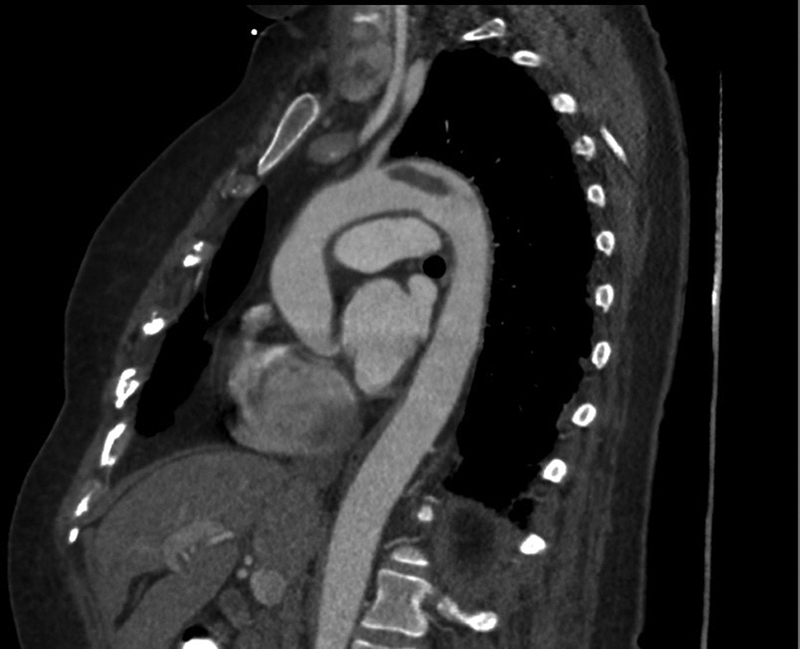
Sagittal reformatted arterial-phase chest computed tomography scan centered on the thoracic aorta. The floating thrombus can be delineated in the aortic arch, distal from the origin of the left subclavian artery.

**Fig. 3:**
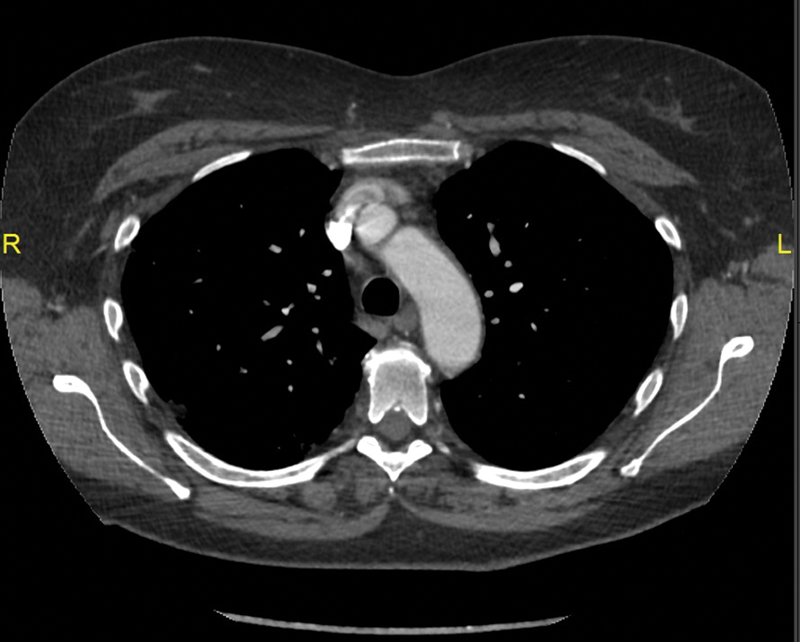
Follow-up axial arterial-phase chest computed tomography scan 2 weeks posttreatment shows no residual thrombus in the aortic arch.

**Fig. 4:**
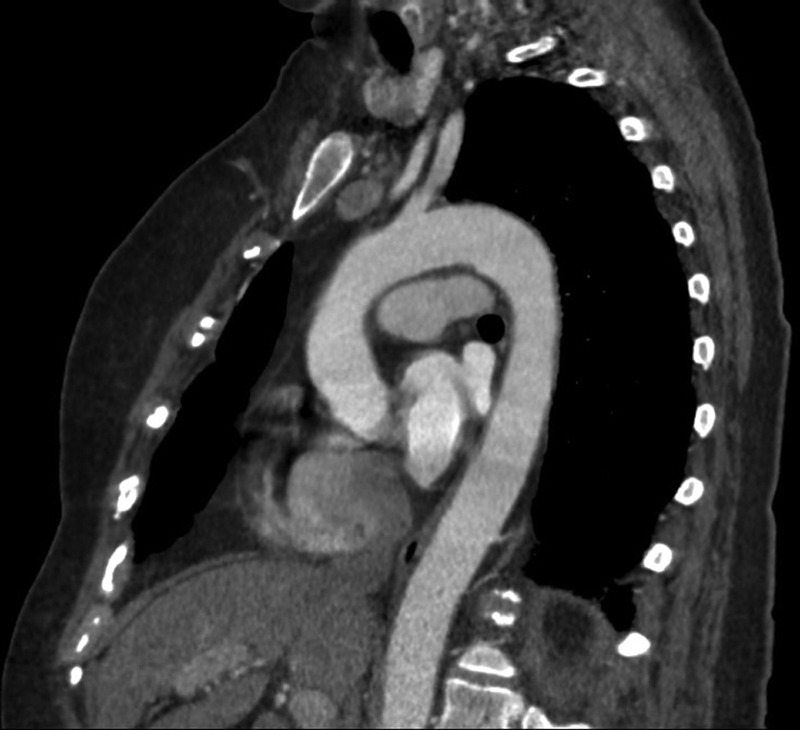
Follow-up sagittal reformatted arterial-phase chest computed tomography scan 2 weeks posttreatment shows no residual thrombus in the aortic arch.

A 70-year-old woman with a previous history of stage-I cervical cancer was referred to the department of thoracic and vascular surgery after a floating thrombus was found in the distal aortic arch. The thrombus was seen on a contrast-enhanced computed tomography (CT) scan which was performed to rule out a possible paraneoplastic giant-cell arteritis. The CT scan showed an aortic mural thrombus (AMT) starting in the aortic arch with extension into the proximal descending aorta ( Figs. 1 and 2 ). There were no signs of embolization clinically or on imaging. In addition, multiple para-aortic and iliac lymphadenopathies were seen on the CT scan as well. Further medical history did not reveal any previous thrombotic events or a prothrombotic state apart from the history of cervical carcinoma. Medical treatment with lifelong aspirin and therapeutic low–molecular weight heparin (LMWH) was started after CT diagnosis. Moreover, the patient was transferred to the intensive care unit for continuous blood pressure monitoring during 24 hours. During follow-up at 2 weeks and 6 months after diagnosis, no signs of the AMT were visible on the chest CT scan ( Figs. 3 and 4 ). The treatment with LMWH was discontinued after 6 weeks due to a complete resolution of the thrombus on the chest CT scan and negative thrombophilia screen tests. Further follow-up did not show any new thrombotic events in the following period. However, shortly afterward, the patient underwent a positron emission tomography CT scan and was diagnosed with metastasized cervical cancer for which she was treated with chemotherapy.

## Discussion

There are several possible treatment modalities for a floating AMT; however, there is no consensus on which treatment method is the most suitable due to the rarity of the disease. Surgery, thrombolysis, endovascular stenting, and conservative treatment with anticoagulation are all possible modalities that have been reported in the literature. An initial treatment with anticoagulation is generally started, while surgery or endovascular repairs are reserved for patients who do not respond to medical treatment. 4 When a conservative treatment is initiated, strict follow-up with regular ultrasound (US) examinations is necessary for evaluating possible thrombus persistence or peripheral embolization. 5 Although many authors agree that anticoagulation is a safe treatment option, there is no clear consensus on the recommended duration of treatment. Proposed durations vary between complete resolution of the aortic thrombus to life-long anticoagulating treatment. 1

Surgical treatment for a floating AMT usually consists of a thrombectomy or a segmental aortic resection. The latter is usually reserved for malignancies of the aortic wall causing thrombus formation. Endovascular treatment with aortic stent grafts is another possible treatment modality for aortic mural thrombosis. Several cases in the literature have described the use of endovascular stents and have shown favorable outcomes. A possible complication of an endovascular approach is peripheral embolization during the placement of guidewires and the deployment of aortic grafts. However, no such cases have yet been published in the current literature. 1 6

No clear guidelines or consensus exist regarding the optimal treatment modalities for floating AMT. Conservative treatment with LMWH and oral therapeutic coagulation seems to be a valid treatment modality in patients who do not have a high risk of peripheral embolization. LMWHs were used for this patient due to its ease of monitoring and lower bleeding risks compared with vitamin-K antagonists. However, strict follow-up with CT imaging and US checkups at regular intervals is necessary. Often endoscopic US (EUS) examinations are needed due to the fact that the thrombi are difficult to visualize with transthoracic ultrasonography. Surgical and endovascular treatments can be reserved for patients with contraindications to anticoagulation or that do not show thrombus resolution on follow-up ultrasound after therapeutic anticoagulation with LMWH. 1 5 7

Our patient was treated conservatively with oral anticoagulation and strict follow-up with US examinations and chest CT scans. Follow-up after 6 months showed a complete resolution of the thrombus. This case demonstrated that conservative treatment for an AMT is indeed possible in select patients with no contraindications for anticoagulation therapy.
